# Influence of spent ginger yeast cultures on the production performance, egg quality, serum composition, and intestinal microbiota of laying hens

**DOI:** 10.5713/ab.21.0514

**Published:** 2022-03-02

**Authors:** Junhan Liu, Yuhong Jin, Junhua Yang

**Affiliations:** 1 Key Laboratory of Food Processing Technology and Quality Control in Shandong Province, College of Food Science and Engineering, Shandong Agricultural University, Tai’an 271018, China; 2 Institute for Agri-Food Standards and Testing Technology, Shanghai Academy of Agricultural Sciences, Shanghai 201100, China

**Keywords:** Blood Biochemical, Egg Quality, Intestinal Microbiota, Laying Hens, Laying Rate, Spent Ginger Yeast Cultures

## Abstract

**Objective:**

Spent ginger is a byproduct of juice extraction from the rhizome of ginger (*Zingiber officinale*). Despite its nutritional value, it is difficult to preserve or further process and thus is often wasted. This study uses spent ginger as a substrate for fermentation and cultivates spent ginger yeast cultures (SGYCs) that are then added to the feed of laying hens. The effects of SGYCs on production performance, egg quality, serum composition, and intestinal microbiota of laying hens were investigated.

**Methods:**

Eighty 60-week-old Hy-Line Brown hens were separated into 5 experimental groups with 4 replicates per group (4 hens per cage, 4 cages per replicate). The control group was fed a basal diet while experimental groups were also given SGYCs at the levels of 5, 10, 20, and 40 g/kg for 6 weeks.

**Results:**

The addition of SGYCs significantly increased the laying rate and nutrient digestibility, decreased feed conversion ratio, and enhanced the color of egg yolks (p<0.05). No changes were observed in activity levels of alanine aminotransferase and aspartate aminotransferase in the serum (p>0.05), but the activities of superoxide dismutase, glutathione peroxidase, and peroxidase all significantly increased, and contents of malondialdehyde were significantly reduced (p<0.05). In addition, changes in the relative abundance of Firmicutes and Bacteroidetes might be the main factor contributing to the significant increase in the apparent digestibility of crude protein and crude fat in laying hens (p<0.05).

**Conclusion:**

The current evidence shows that dietary supplementation of SGYCs to the feed of laying hens can improve laying rates, enhance antioxidative defenses, and influence dominant intestinal bacteria.

## INTRODUCTION

Eggs are considered an ideal food for humans in terms of nutritional value. Egg protein has amino acid composition highly suitable and can be easily absorbed by the human body, with utilization rates up to 98% [1]. Eggs are also rich in lecithin, sterols, and various micronutrients [2]. The egg industry is of significant economic importance, with over 70 million tons produced annually across the world [3]. Increasing the output and quality of egg production remains of high interest in agricultural research.

Supplementation to the diet of laying hens is a common strategy to improve egg produc tion. Yeast cultures (YCs), which typically refer to mixtures of both yeast (*Saccharomyces cerevisiae* [*S. cerevisiae*]) and the solid or liquid mediums in which they were fermented, are considered high-quality supplements because they contain various bioactive substances including yeast cells, vitamins, amino acids, organic acids, and oligosaccharides [4]. Recent research has shown that the addition of YCs in poultry feeds often has positive effects. Gómez et al [5] demonstrated that YCs supplements improved feed conversion ratio, breast yield, and ileal digestibility of dry matter in broiler chicks. Ma et al [6] have shown that the addition of distiller’s grain based YCs to the diet of broiler chicks results in rapid increases in body weight, and Yalçin et al [7] showed that inclusion of commercial YCs in the diet of laying hens can decrease cholesterol levels in the yolk while also increasing the overall weight of the eggs.

Spent ginger is a byproduct of commercial processes that crush the rhizome of ginger (*Zingiber officinale*) to extract juices. Due to the high moisture content in spent ginger, it is difficult to preserve or further process, and thus often discarded. However, it is also an ideal substrate to culture YCs for feed supplementation. Large molecules such as proteins and starches in spent ginger can be broken down into by yeast, which is conducive to absorption. As well, ginger contains various monoterpenes (cineole, citral, limonene, and *α/β*-pinenes), sesquiterpenes (*β*-elemene, farnesene and zerumbone), phenols (gingerols, [6]-shogaol, [6]-paradol and zingerone), and diphenylheptane compounds [8], known to have various health benefits including anti-inflammatory [9], anti-oxidization [10], anti-bacterial [11], blood sugar reducing [12], and gastrointestinal protecting effects [13]. Yeast cultures fermented in spent ginger are therefore expected to have various nutritional and medicinal benefits, making them a valuable feed additive.

Currently, research regarding the cultivation of YCs using spent ginger as a substrate, or the application of such cultures as a feed additive, is lacking. The present study aims to evaluate how the addition of spent ginger yeast cultures (SGYCs) to the diet of laying hens may affect their blood indicators and intestinal bacteria, as well as their productivity and the quality of eggs produced. We also explore the potential mechanisms through which SGYCs influence the output and quality of egg production. Our work aims to reduce the waste of ginger byproducts and promote the sustainable development of animal husbandry.

## MATERIALS AND METHODS

### Animal care

The present experiment was reviewed and approved by the Institutional Animal Care and Use Committee at Shanghai Academy of Agricultural Sciences (SAASPZ0920001).

### Materials

Hy-Line Brown laying hens (60 weeks of age) were provided by Jun’an Egg Farm (Shanghai, China). Spent ginger yeast cultures were provided by the Fermentation Engineering Laboratory of Shandong Agricultural University (Tai’an, China). Cultivation procedures were as follows: spent ginger was mixed with water at a 1:3 ratio, to which sucrose was added at 102 g/L and *S. cerevisiae* (WET 137; Eaton, Nettersheim, Germany) at 0.4 g/L. The mixture was fermented in a semi-solid state at 20°C until sugar contents were less than 4 g/L and no longer changed for two consecutive days, then squeezed with a plate and frame press. The pulp was dried at 55°C and crushed. The proximate composition of SGYCs was as follows: 9.6% moisture, 9.8% crude protein, 5.2% crude fat, 24.5% crude fiber, 6.0% ash, and 45.0% total crude carbohydrates.

### Animals, diets, and experimental design

A total of 80 Hy-Line Brown laying hens were randomly allocated to one of five treatment groups. Each treatment had 4 replicates with 4 hens per replicate. The basal diet of the five treatment groups were supplemented with SGYCs at levels of 0 (control), 5, 10, 20, and 40 g/kg. All experimental diets were approximately isonitrogenous and isocaloric and were formulated to meet or exceed nutrient requirements recommended by the NRC [14]. Ingredients and nutrient composition of the basal diet are shown in Table 1.

The study was conducted for 8 weeks, including 2 weeks of adaptation and 6 weeks of the experiments. The laying hens were housed in an environmentally controlled room where the temperature was 20°C±2°C. A 16-h lighting schedule (16 light:8 dark) was used during the entire experiment. Hens were raised in cages: each cage was 400 mm long, 450 mm deep, 450 mm high in the front and 380 mm high in the rear. The bottom of the cage leaned 7 degrees and was equipped with a nipple drinker and a trough feeder. Each cage had 4 laying hens, which could eat and drink freely. Feeding, egg collection, and cleaning of the coop were performed daily. Feces were cleaned and the chicken coop was sprayed and disinfected once a week.

### Laying performance and egg quality traits

During the feeding trial, the total number of eggs and total egg weight of each replicate were recorded every day. Feed consumption of each replicate was recorded each week. The average egg weight (g), laying rate (%), average daily feed intake (g), and feed conversion ratio of each replicate were calculated at 3-week intervals. The average egg weight was the ratio of the total number of eggs to the total egg weight. The laying rate refers to the number of hens that laid eggs as a percentage of the total number of laying hens. The feed conversion ratio was calculated by dividing the weight of feed consumed by the weight of eggs produced.

To determine egg quality, 12 eggs were randomly selected from each group on day 21 and day 42 of the feeding experiment. The major and minor axes of the eggs were measured using the egg shape index meter (FHK, Tokyo, Japan), and the egg shape index (long diameter/short diameter of eggs) was calculated. An egg quality tester (DET-6000; Nabel, Kyoto, Japan) was used to determine eggshell hardness (kgf), yolk color, albumen height (mm) and the Haugh unit. The determination of yolk color value was based on the “Roche color fan (RCF)” system, which consists of 15 color samples corresponding to values 1 to 15. The thickness of the shell at the blunt end, tip and middle of the egg was measured using an eggshell thickness tester (C1012XBS; Mitutoyo corp, Kanagawa, Japan) and used to calculate average eggshell thickness.

### Apparent digestibility of nutrients

Apparent digestibility was determined using the total feces collection method [15]. After the end of the feeding experiment period, 8 laying hens were randomly selected from each group to be unfed for 2 days to empty the original feces in the intestines, then re-fed for 2 days and had their feces collected for 4 consecutive days after feeding. Feathers and other foreign objects were removed from the collected feces, after which the feces were dried to a constant weight in an oven at 60°C and then pushed through a sieve (0.425 mm) using a sealed pulverizer (XL-20B; Xulang Machinery, Guangzhou, China). Crude fat (No. 920.39), crude protein (No. 978.04), crude fiber (No. 978.10), and ash (No. 930.05) were determined following methods of the Official Society of Analytical Chemists [16]. Apparent nutrient digestibility was calculated as follows:


Nutrient digestibility=[(nutrient intake-nutrient voided)/(nutrient intake)]×100%

Nutrient intake=nutrient in diet×feed intake

Nutrient voided=nutrient in feces×amount of feces voided

### Routine blood indices and serum biochemical parameters

On the 42nd day of the experimental period, 12 laying hens were randomly selected from each treatment group of 16. Blood samples were collected from the wing vein of each hen using disposable needles, and then placed in a vacuum sealed sampling vessel. A blood cell analyzer (MEK-6400C; Celltacɑ, Tokyo, Japan) was used to determine routine indices in the whole blood, including red blood cells (RBC), hemoglobin (HGB), hematocrit (HCT), mean corpuscular volume (MCV) and platelet count (PLT). Blood biochemical indicators were determined using the serum. Blood samples were centrifuged (15 min, 3,000 rpm) at 4°C in a centrifuge (AG 22331; Hamburg, Eppendorf, Germany) to obtain serum samples, which were stored at −80°C. The following biochemical indicators were determined using an automatic biochemical analyzer (AU5800; Beckman Coulter, Brea, CA, USA): glucose, alanine aminotransferase (ALT), aspartate aminotransferase (AST), total protein (TP), and total cholesterol (CHOL).

### Serum antioxidant capacity

The serum samples prepared in the previous section were used to determine the serum antioxidant capacity. The contents of glutathione peroxidase (GSH-Px), superoxide dismutase (SOD), peroxidase (CAT), and malondialdehyde (MDA) in the serum were determined using a microplate reader (Multiskan Sky; Thermo Fisher, Waltham, MA, USA) and a Beyotime Biotechnology testing kit (Beyotime Biotechnology, Shanghai, China), following manufacturer’s instructions.

### Analysis of intestinal microbiota of laying hens

On the 42nd day of the experimental period, 4 laying hens were randomly selected from each group to collect fresh feces. The fresh feces were placed in a sterile cryotube and placed in liquid nitrogen for rapid freezing, then sent to the laboratory to be stored in at −80°C for subsequent analyses. Fecal samples were separately iced and homogenized, and each was processed using a QIAamp DNA Stool Mini Kit (Qiagen, Hilden, Germany) in strict accordance with the manufacturer’s standard procedures to extract microbial genomic DNA. Primers 338F (5′-ACTCCTACGGGAGCAG CA-3′) and 806R (5′-GGACTACHVGGGTWTCTAAT -3′) were used for polymerase chain reaction amplification to detect V3–V4 regions of bacterial 16S rRNA. Sequencing and bioinformatics analysis were performed by PersonalBio Ltd. (Shanghai, China). Briefly, paired-end sequencing of community DNA fragments was conducted on the Illumina Miseq platform, and the original sequencing data was saved in FASTQ format. Sequences were quality filtered, denoised, merged, and had chimeras removed using the DADA2 plugin [17]. Sequences from all samples were sorted by abundance, after which they were used to select operational taxonomic units (OTUs) at a threshold of 97% similarity. Finally, the output of the representative sequences and OTUs were organized into a table. The Greengenes Database (Release 13.8) was used to classify the representative sequences of each OTU. Based on results from taxonomic classification of the OTU representative sequences, the composition table of the microbiota community in each sample at each classification level was summarized and visualized with a stacked bar chart.

### Statistical analysis

Statistical analyses were performed using SAS 9.2. A one-way analysis of variance was performed to detect significance differences among groups. Means were also compared using Tukey’s multiple comparison test to determine pair-wise differences. The linear and quadratic responses were analyzed using orthogonal polynomials. Differences at p<0.05 were considered significant, whereas p-values between 0.05 and 0.1 were interpreted as trends. The Spearman rank correlation coefficient calculated in IBM SPSS Statistics 25 was used to analyze correlations, with p<0.05 indicating significant correlation and p<0.01 indicating extremely significant correlation.

## RESULTS AND DISCUSSION

### Laying performance and egg quality traits

As seen in Table 2, while SGYC supplementation had no significant effects on average egg weight or average daily feed intake (p>0.05), it had a significant effect on the laying rate and feed conversion ratio. In the latter half of the experiment, the relationship between laying rate and SGYC supplementation followed a quadratic curve (p = 0.022), with laying rate first increasing and then decreasing with increased SGYC supplementation. In addition, the laying rate in hens given 10 g/kg supplements (85.1%) was significantly higher than the control group (74.3%) throughout the entire experiment period (p<0.05). Throughout both halves of the experimental period, increasing supplementation of SGYCs also led to first decreases and then increases in the feed conversion ratio, their relationship following a quadratic curve (p = 0.007). Moreover, SGYC supplementation had significant effects on the feed conversion ratio (p<0.05). Specifically, the 10 g/kg supplemental group had distinctly lower feed conversion ratio compared to the control. This result may be linked to the increase in egg weight: on average, eggs from the 10 g/kg supplemental group weighed 0.23 g more compared to the control group. These results indicate that the addition of SGYCs to feeds for laying hens can effectively increase the frequency of egg laying and lower feed conversion ratio, and that such effects manifest in a gradual manner. Moreover, our results suggest that optimal effects are obtained when supplements are added at 10 g/kg of feed. These results corroborate with previous studies such as Zhang et al [18], who used corn germ meal and wheat bran as substrates, fermented them into YC with *S. cerevisiae*, and found that increased egg-laying rate and reduced feed conversion ratio when a 0.3% supplementation of *S. cerevisiae* cultures was added to feeds.

Although SGYC supplementation did not significantly change the properties of the eggshell including shell strength, shell thickness, and shape index, it had a significant effect on the yolk color. Analysis of eggs collected on the 42nd day of the experimental period showed that yolks of eggs laid by hens from the treatment groups were significantly different in color compared to the control (p<0.01), and the RCF values of yolk color obtained from the 40 g/kg group were as high as 8.90. The color of egg yolks is known to be a key factor in the marketing and sales of eggs, and golden or orange yolks with RCF>8.0 are typically more preferred by consumers [19]. Research has shown that most fat-soluble pigments can affect the color of egg yolks. Pigments responsible for the yellow color are typically carotenes [20], and most commonly xanthophylls (C_40_H_56_O_2_). Common pigments present in feeds include lutein, zeaxanthin, and cryptoxanthin [21]. The enhanced yellow color in egg yolks from this study could be attributed to curcumin (C_21_H_20_O_6_) found in ginger rhizomes. Curcumin is an isomer of lutein with very similar properties. Park et al [22] have also reported that the addition of 0.5% curcumin resulted in egg yolks with enhanced color.

### Apparent digestibility of nutrients

The apparent digestibility of nutrients in laying hens are as shown in Table 3. Crude protein, crude fat, crude fiber, and ash all increased first and then decreased with increasing supplementation of SGYCs. Among them, the addition of SGYCs had a significant effect on the apparent digestibility of crude protein and crude fats in the feed (p<0.01), and the 10 g/kg supplement group had the highest apparent digestibility for all nutrients. This can be explained by several known properties of ginger. Active ingredients in ginger have been shown to antagonize muscarinic and histaminergic receptors and have a beneficial effect in facilitating gastrointestinal movement [23]. Studies have also reported that ginger can improve the activities of digestive enzymes such as lipase, sucrase and protease and thus facilitate digestion in the intestines [24]. As well, Zhang et al [18] demonstrated the effects of YCs in improving the digestion of laying hens by showing that adding *S. cerevisiae* cultures to the diet of laying hens can increase activities of digestive enzymes in the chyme while also decreasing the toxin levels in their plasma.

In addition, results of the present study show that during the latter half of the experimental period (day 21 to 42), the laying rate was significantly positively correlated with the apparent digestibility of crude fat, crude fiber, crude protein, and ash (p<0.05). Therefore, we conclude that the apparent digestibility of nutrients is closely related to the laying rate. Other studies have also shown that increasing the digestibility of nutrients in laying hens can improve rates of egg production [25].

### Routine blood indices and serum biochemical components

Alterations in hematological indices including the decrease of RBC, HGB, and HCT are the main symptoms of anemia [26]. As shown in Table 4, the addition of SGYCs had no significant effect on blood indicators including RBC, MCV, and PLT (p>0.05). However, HGB and HCT levels followed a quadratic curve with increasing supplementation of SGYCs (p<0.05). This phenomenon indicates that SGYCs may have the potential to alleviate anemia, although requiring further research to elucidate. Results of this study also showed that there were no significant differences in the activities of ALT and AST in the serum between the groups (p>0.05). These two enzymes are excreted into the circulatory system by damaged liver cells, and thus their increased activity in the serum is a reliable indicator of liver damage or dysfunction [27]. The lack of differences in their activities in the present study suggests that the addition of SGYCs had no adverse effects on liver function.

As nutrients enter the blood after being absorbed and converted, the increased digestibility of nutrients in hens fed SGYCs is also reflected in nutrient levels in the serum. The results revealed that TP and CHOL content in the serum were significantly positively correlated with the apparent digestibility of proteins and fats, respectively (p<0.01). Higher protein content in the serum is in turn correlated with egg albumen height (p<0.05). As albumen height is a main indicator in evaluating egg quality that reflects both nutrient levels and egg white consistency, the results also corroborate previous research that serum protein content is a key factor in determining egg quality [28].

### Serum antioxidant capacity

As a byproduct of their metabolism, laying hens produce superoxide anions (O_2_^•−^), which are cytotoxic free radicals that can attack the polyunsaturated fatty acids in the biofilm, causing lipid oxidations and generating MDA [29]. MDA will then cause the cross-linking between membrane lipids and membrane proteins and ultimately disturb normal cell metabolism. SOD, GSH-Px, and CAT are the main endogenous antioxidant enzymes in animal cells and work in coordination to eliminate free radicals such as O_2_^•−^ [30]. Thus, the concentration of these molecules are indicators of cellular health and function.

**Figure 1:**
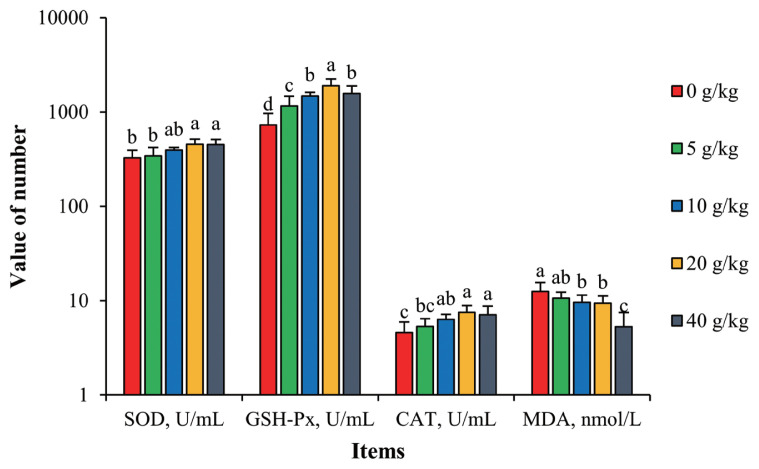
Effects of SGYCs on serum antioxidant capacity in laying hens. SGYCs, spent ginger yeast cultures; SOD, superoxide dismutase; GSH-Px, glutathione peroxidase; CAT, peroxidase; MDA, malondialdehyde. Values are presented as means±standard error. ^a–d^ Columns with different lowercase letters differ significantly (p<0.05).

As seen in Figure 1, with increased supplementation of SGYCs, activities of SOD, GSH-Px, and CAT all increased, and correspondingly levels of MDA decreased. Hens given 20 g/kg supplementation had the highest enzymatic activities and all were significantly different compared to the control (p<0.05). On the other hand, the treatment group given 40 g/kg supplementation had the lowest levels of MDA, up to 28.4% lower than the control (p<0.05). These results show that SGYCs can facilitate anti-oxidizing reactions in hens, lowering levels of MDA and thus reducing physiological abnormalities. Similar observations have been made by Zhao et al [31], who added powdered ginger to the feed of laying hens. These effects can be attributed to the various antioxidants found in ginger, such as [6]-gingerol, which inhibits lipid oxidation [32], or flavonoids, which can remove free radicals such as hydroxyls and superoxide anions.

### Intestinal microbiota

**Figure 2:**
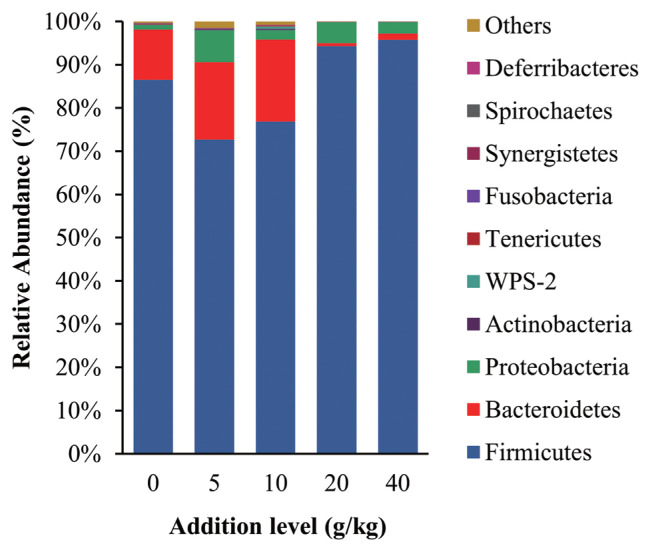
Compositional changes in the intestinal microbiota of laying hens at the phylum levels.

The intestinal microbiota community structure is shown in Figure 2, the dominant phyla of gut bacterial were Firmicutes, Bacteroidetes, and Proteobacteria in all treatment groups, which are the same groups reported to be most dominant in the intestinal tract of healthy poultry [33]. Thus, it appears that the addition of SGYCs did not adversely affect the intestinal microbiota of laying hens. Firmicutes was the most dominant phylum in all treatment groups and are known for degrading carbohydrates in the intestine. Considering that carbohydrates are the main source of energy for all organisms, it is unsurprising that Firmicutes have a great advantage. These observations are also consistent with previous reports such as those of Shang et al [27]. Additionally, studies have reported that there are more protein-degrading bacteria species in Bacteroidetes [34]. In this study, the relative abundance of Bacteroidetes was higher in the 10 g/kg supplement group when compared with the control group (19.01% vs 11.68%), which may explain the higher apparent digestibility of proteins in the groups given 10 g/kg supplements, although these changes were not significant (p>0.05). It has also been reported that ingredients including polyphenols, flavonoids, and dietary fiber changed the relative abundance of Firmicutes and Bacteroidetes in the intestine [35,36], and SGYCs are rich in these ingredients. Additionally, the ratio of Firmicutes to Bacteroidetes is known to be negatively correlated with fat metabolism [37]. The results of the present study also follow this pattern: the 10 g/kg supplement group had the lowest Firmicutes to Bacteroidetes ratio (4.04) and the highest apparent fat digestibility (87.68%), which is followed by the 5 g/kg supplement group with Firmicutes to Bacteroidetes ratio of 4.06 and fat apparent digestibility of 85.81%. The above results are also concurrent with Liu et al, who found that the improvement of various production aspects in laying hens given commercial YCs, including the quality of eggs, immune function, antioxidant capacity, reproduction efficiency, digestion, and absorption capacity, were linked to the change in the relative abundance of gut microbiota [38].

## CONCLUSION

The current evidence shows that dietary supplementation of SGYCs to the feed of laying hens can improve laying rates and decrease feed conversion ratio, and such effects are the most prominent when they are added at 10 g/kg of feed. Eggs laid by treatment group hens also had yolks with enhanced color. Additionally, enzymatic activity of antioxidants in the serum also significantly increased, suggesting a lower occurrence of physiological abnormalities. These findings all support the potential application of SGYCs as a feed supplement that benefits laying hens. The potential mechanisms through which SGYCs influence production performance and egg quality of laying hens are summarized in Supplementary Figure S1: the addition of SGYCs firstly changes the relative abundance of dominant microbiota in the intestines of laying hens. This improves the apparent digestibility of nutrients in the feed, which promotes egg laying and increases the content of nutrients in the blood, especially the TP content. The increased protein content in the serum also improves protein quality of the eggs.

## Figures and Tables

**Table 1:** Ingredients and nutrient composition of basal diet (g/kg as fed unless noted)

Item	Content
Ingredients	
Corn	600.0
Soybean meal	210.0
Soybean oil	15.0
CaHPO_4_	10.0
Limestone	80.0
Fish meal	20.6
Wheat bran	50.0
NaCl	3.2
DL-Methionine	1.2
Premix^1)^	10.0
Nutrient composition	
Crude protein	161
Crude fat	25
Crude fiber	40
Ash	120
Calcium	33.5
Total phosphorus	7.0
Lysine	9.8
Methionine+cysteine	8.0
ME (Kcal/kg)	2,850

ME, metabilizable energy.

1) Supplied per kilogram of diet: vitamin A, 12,000 IU; vitamin D_3_, 4,000 IU; vitamin E, 30 IU; vitamin K_3_, 4 mg; vitamin B_6_, 5 mg; vitamin B_12_, 0.1 mg; thiamine, 2 mg; riboflavin, 8 mg; niacin, 45 mg; biotin, 0.2 mg; pantothenic acid, 11 mg; folic acid, 2 mg; Mn, 80 mg; Fe, 100 mg; Zn, 80 mg; Cu, 8 mg; I, 0.8 mg; Se, 0.3 mg.

**Table 2:** Effects of SGYCs on production performance^1)^ and egg quality^2)^ in laying hens

Items	Additive concentration (g/kg)					SEM	p-value^*^		
	
	0	5	10	20	40		ANOVA	Linear	Quadratic
Laying rate (%)									
Day 0 to 21	76.2	81.0	86.9	82.2	82.1	1.846	0.546	0.332	0.270
Day 21 to 42	72.3	78.9	83.3	77.9	75.3	1.373	0.075	0.618	0.022
Day 0 to 42	74.3^b^	79.9^ab^	85.1^a^	80.0^ab^	78.7^ab^	1.181	0.049	0.287	0.021
Average daily feed intake (g)									
Day 0 to 21	106.2	106.2	106.2	106.2	106.2	0.016	0.977	0.689	0.876
Day 21 to 42	106.3	106.3	106.2	106.3	106.2	0.004	0.171	0.398	0.549
Day 0 to 42	106.2	106.2	106.2	106.2	106.2	0.009	0.918	0.549	0.830
Feed conversion ratio									
Day 0 to 21	2.29	2.14	1.96	2.07	2.08	0.054	0.505	0.236	0.217
Day 21 to 42	2.35	2.18	2.04	2.09	2.29	0.041	0.049	0.463	0.007
Day 0 to 42	2.32^a^	2.16^ab^	2.01^b^	2.08^ab^	2.19^ab^	0.034	0.037	0.151	0.007
Average egg weight (g)									
Day 0 to 21	61.21	62.17	61.88	61.02	62.09	0.223	0.344	0.705	0.919
Day 21 to 42	62.53	61.67	62.33	61.56	61.75	0.187	0.434	0.228	0.458
Day 0 to 42	61.87	61.91	62.10	61.29	61.92	0.146	0.477	0.612	0.874
Albumen height (mm)									
Day 21	6.93	7.13	7.41	7.33	7.35	0.101	0.580	0.154	0.260
Day 42	7.62	7.64	7.87	7.80	7.46	0.103	0.746	0.826	0.489
Yolk color (RCF)									
Day 21	7.87	7.69	8.29	8.14	8.19	0.082	0.137	0.070	0.178
Day 42	7.47^b^	8.66^a^	8.87^a^	8.41^a^	8.90^a^	0.069	<0.01	<0.01	<0.01
Haugh unit									
Day 21	82.43	83.49	85.30	85.01	84.73	0.601	0.543	0.154	0.234
Day 42	86.00	87.37	87.94	87.66	85.40	0.545	0.506	0.811	0.197
Shell strength (kgf)									
Day 21	3.79	3.71	3.97	3.59	3.93	0.077	0.511	0.767	0.907
Day 42	4.37	4.13	4.41	4.23	4.34	0.067	0.678	0.941	0.909
Shell thickness (mm)									
Day 21	0.35	0.35	0.35	0.34	0.36	0.003	0.300	0.787	0.559
Day 42	0.38	0.38	0.39	0.38	0.38	0.003	0.850	0.833	0.903
Shape index									
Day 21	1.31	1.30	1.31	1.31	1.30	0.004	0.756	0.808	0.803
Day 42	1.31	1.30	1.30	1.30	1.30	0.004	0.981	0.675	0.841

SGYCs, spent ginger yeast cultures; SEM, standard error of the means; ANOVA, analysis of variance.

1) Data are means for 4 replicates of 4 laying hens/replicate.

2) Data are means for 4 replicates of 3 eggs/replicate.

a,b The means within a row with different lowercase letters are significantly different (p<0.05).

* Responses (linear or quadratic) to different levels of SGYCs in the diet of laying hens.

**Table 3:** Effects of SGYCs on apparent digestibility of nutrients in laying hens

Items	Additive concentration (g/kg)					SEM	p-value^*^		
	
	0	5	10	20	40		ANOVA	Linear	Quadratic
Crude fat (%)	83.92^ab^	85.81^a^	87.68^a^	84.48^ab^	80.08^b^	0.740	0.007	0.109	<0.01
Crude fiber (%)	51.10	53.64	54.64	51.39	50.57	0.674	0.086	0.788	0.042
Crude protein (%)	58.37^b^	62.20^ab^	63.42^a^	60.98^ab^	58.22^b^	0.675	0.009	0.755	<0.01
Ash (%)	45.68	46.40	47.59	45.98	44.85	0.321	0.056	0.376	0.019

SGYCs, spent ginger yeast cultures; SEM, standard error of the means; ANOVA, analysis of variance.

Data are means for 4 replicates of 2 laying hens/replicate.

a,b The means within a row with different lowercase letters are significantly different (p<0.05).

* Responses (linear or quadratic) to different levels of SGYCs in the diet of laying hens.

**Table 4:** Effects of SGYCs on blood routine indices and serum biochemical parameters in laying hens

Items	Additive concentration (g/kg)					SEM	p-value^*^		
	
	0	5	10	20	40		ANOVA	Linear	Quadratic
RBC (×10^6^ μL)	2.50	2.59	2.60	2.70	2.59	0.028	0.322	0.172	0.179
HGB (g/dL)	13.76	14.48	14.60	15.15	14.48	0.159	0.111	0.067	0.039
HCT (%)	29.48	30.40	31.30	31.73	26.69	0.316	0.093	0.437	0.035
MCV (fL)	115.40	116.90	117.50	117.80	116.00	0.566	0.647	0.602	0.301
PLT (×10^3^ μL)	14.11	14.00	14.44	14.56	14.00	0.756	0.999	0.953	0.982
GLU (mmol/L)	12.09	11.31	11.53	11.50	11.44	0.236	0.879	0.515	0.681
ALT (U/L)	1.1	1.1	1.0	1.1	1.1	0.137	0.997	1.000	0.970
AST (U/L)	196.43	194.57	187.14	203.29	206.00	3.137	0.361	0.218	0.219
TP (g/L)	52.37	53.89	54.49	52.83	49.13	0.827	0.314	0.174	0.089
CHOL (mmol/L)	2.33	2.70	2.79	2.34	2.32	0.096	0.332	0.581	0.215

SGYCs, spent ginger yeast cultures; SEM, standard error of the means; ANOVA, analysis of variance; RBC, red blood cells; HGB, hemoglobin; HCT, hematocrit; MCV, mean corpuscular volume; PLT, platelet count; GLU, glucose; ALT, alanine aminotransferase; AST, aspartic acid aminotransferase; TP, total protein; CHOL, total cholesterol.

Data are means for 4 replicates of 3 laying hens/replicate.

* Responses (linear or quadratic) to different levels of SGYCs in the diet of laying hens.

## SUPPLEMENTARY MATERIAL

**Supplementary Figure S1**. Potential mechanism of SGYCs affecting egg production and egg quality in laying hens.
