# Supplementary material for: Influence of spent ginger yeast cultures on the production performance, egg quality, serum composition, and intestinal microbiota of laying hens
Source: Anim Biosci. 2022 Mar 2;35(8):1205–14. doi: 10.5713/ab.21.0514 (PMC9262721; doi:10.5713/ab.21.0514)
Supplement: Supplementary file 1 [file ab-21-0514-suppl.pdf]

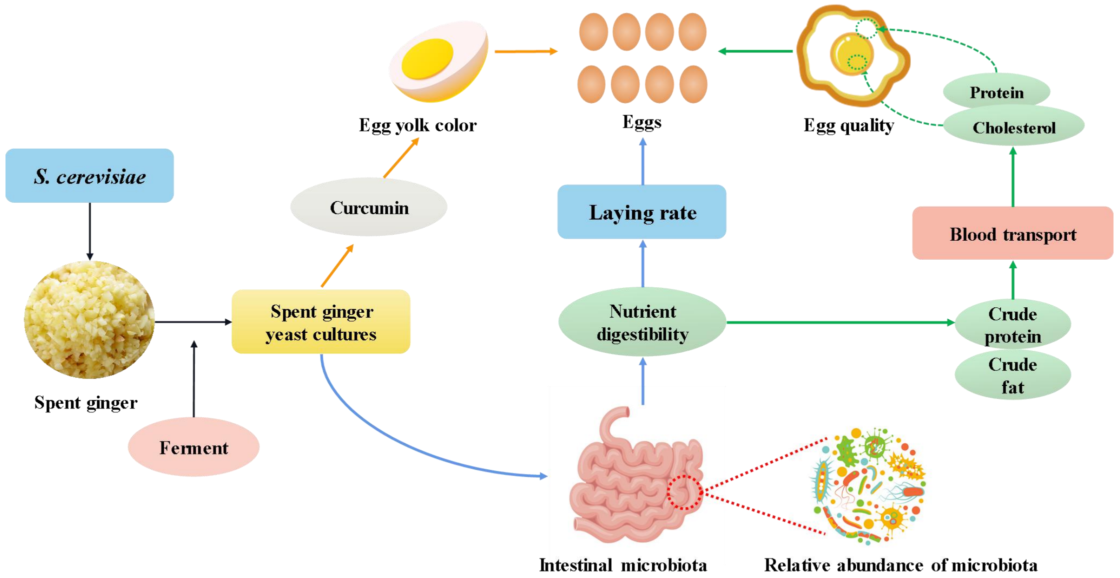

Figure. S1 Potential mechanism of SGYCs affecting egg production and egg quality in laying hens. SGYCs improve nutrient digestibility by affecting the relative abundance of dominant microbiota in the intestines of laying hens, thus improving laying rates and egg quality. SGYCs, spent ginger yeast cultures.
